# Molecular imaging of atherosclerosis with integrated PET imaging

**DOI:** 10.1007/s12350-016-0766-y

**Published:** 2017-01-11

**Authors:** Basma Hammad, Nicholas R. Evans, James H. F. Rudd, Ahmed Tawakol

**Affiliations:** 1 Cardiology Division, Massachusetts General Hospital and Harvard Medical School, Boston, MA USA; 2 Division of Cardiovascular Medicine, University of Cambridge, Cambridge, UK; 3 Integrative Bio-Imaging Program and Cardiac MR PET CT Program, Cardiology Division, Massachusetts General Hospital, 165 Cambridge Street, Suite 400, Boston, MA 02114-2750 USA

**Keywords:** Atherosclerosis, PET/CT imaging, Echocardiography, Myocardial perfusion imaging: SPECT, Magnetic resonance imaging

## Abstract

Atherosclerotic diseases account for nearly half of all deaths and are leading causes of adult disability. Our understanding of how atherosclerosis leads to cardiovascular disease events has evolved: from a concept of progressive luminal narrowing, to that of sudden rupture and thrombosis of biologically active atheroma. In concert with this conceptual shift, contemporary imaging techniques now allow imaging of biological processes that associate with plaque instability: active calcification and plaque inflammation. This review focuses on opportunities provided by positron emission tomography/computed tomography, to identify these high-risk biological features of atherosclerosis.

**Electronic supplementary material:**

The online version of this article (doi:10.1007/s12350-016-0766-y) contains supplementary material, which is available to authorized users.

## Introduction

Despite advances in cardiovascular therapeutics, atherosclerotic events, such as myocardial infarction (MI) and stroke, continue to account for nearly half of all deaths and are a leading cause of adult disability. Decades’ worth of scientific studies refined our understanding of how atherosclerotic disease results in atherothrombotic events: from a concept of progressive luminal narrowing to that of sudden rupture and thrombosis of biologically active atheroma. In concert with this conceptual shift, the approach to imaging atherosclerotic milieu has moved beyond lumenological imaging and now incorporates identification of high-risk features of the arterial wall, including structural features, along with key biological processes (such as active calcification and plaque inflammation).^1^,^2^ This review focuses on opportunities provided by positron emission tomography/computed tomography (PET/CT) to characterize such biological features of high-risk atherosclerotic plaque.

## Atherosclerotic plaque biology^3^

**Figure 1:**
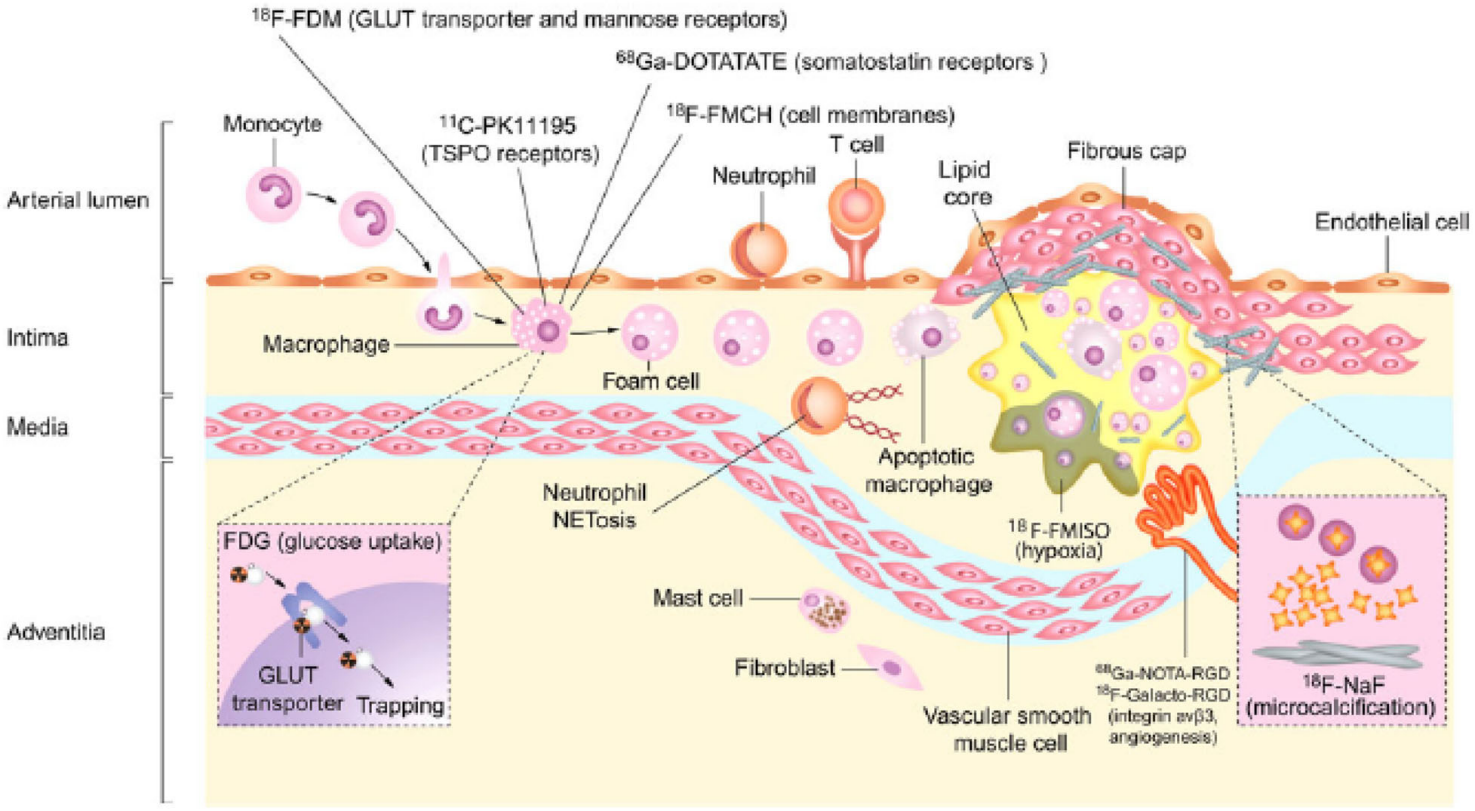
Molecular targets for positron emission tomography atherosclerosis imaging. During atherosclerosis development macrophages avidly utilize glucose, and simultaneously accumulate ^18^F-fluorodeoxyglucose (FDG). ^18^F-fluorodeoxymannose also accumulates in macrophages, entering via glucose transporters and mannose receptors. Somatostatin receptors are also expressed on activated macrophages, and act as a target for the tracer 68 Ga-DOTATATE. Macrophages-mediated inflammation can also be detected by novel tracers targeting translocator protein receptors (^11^C-PK11195) and macrophage cell membranes (^18^F-FMCH). Additional positron emission tomography tracers targeting atherosclerosis can identify microcalcification (^18^F-sodium fluoride), neoangiogenesis (68 Ga-NOTA-RGD, ^18^F-Galacto-RGD), and cellular hypoxia (^18^F-FMISO). Reprinted with permission^1^

Atherosclerotic plaques are biologically active tissues. Oxidized low-density lipoprotein (LDL) particles induce endothelial cells to express leukocyte adhesion molecules (e.g., vascular cell adhesion molecules-1 or VCAM-1, selectins). These adhesion molecules prompt recruitment of circulating monocytes and T-lymphocytes that in turn release cytokines and magnify the inflammatory cascade. Monocytes differentiate into resident macrophages, which phagocytose oxidized LDL and subsequently evolve into foam cells. As inflammatory cells and lipids accumulate within the atheroma, the plaque enlarges. Macrophages and smooth muscle cells undergo apoptosis, and their discarded lipids add to the evolving lipid-rich necrotic core. Neovascularization is triggered by hypoxia and is considered a marker of ongoing disease activity and indicative of a high-risk plaque. These new microvessels, originating from the adventitial vasa vasorum, are immature and leaky resulting in local intra-plaque hemorrhage. Active calcium deposition represents another common feature of vulnerable plaque. Eventually, the production of matrix metalloproteinases by activated leukocytes leads to enzymatic degradation of the plaque’s fibrous cap. This disruption can prompt plaque rupture, leading to atherothrombotic consequences. Accordingly, atherosclerotic plaques provide several attractive targets for imaging biological activity (Figure 1).

## Imaging of vascular inflammation with (FDG-PET/CT)^1^,^4^

**Figure 2:**
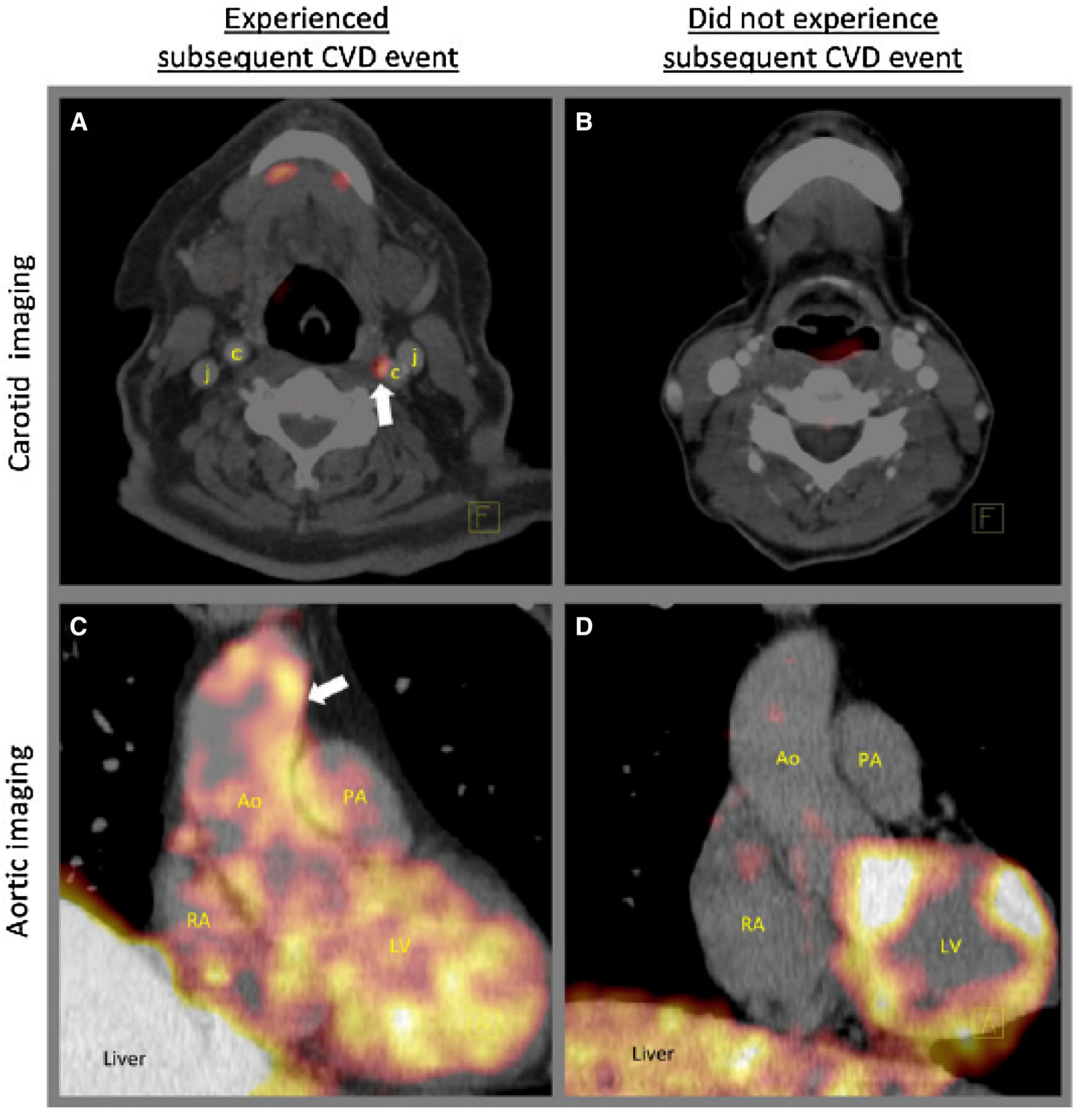
Example positron emission tomography images demonstrating high and low arterial ^18^F-fluorodeoxyglucose uptake in two individuals. One individual, who experienced a subsequent CVD event after imaging, (**A** and **C**), demonstrated high carotid FDG uptake (*white arrow*, **A**) and high aortic FDG uptake (*white arrow*, **C**) prior to that event. In contrast, a second individual who did not experience a subsequent, (**B** and **D**) manifested low arterial FDG uptake. Reprinted with permission^1^

**Figure 3:**
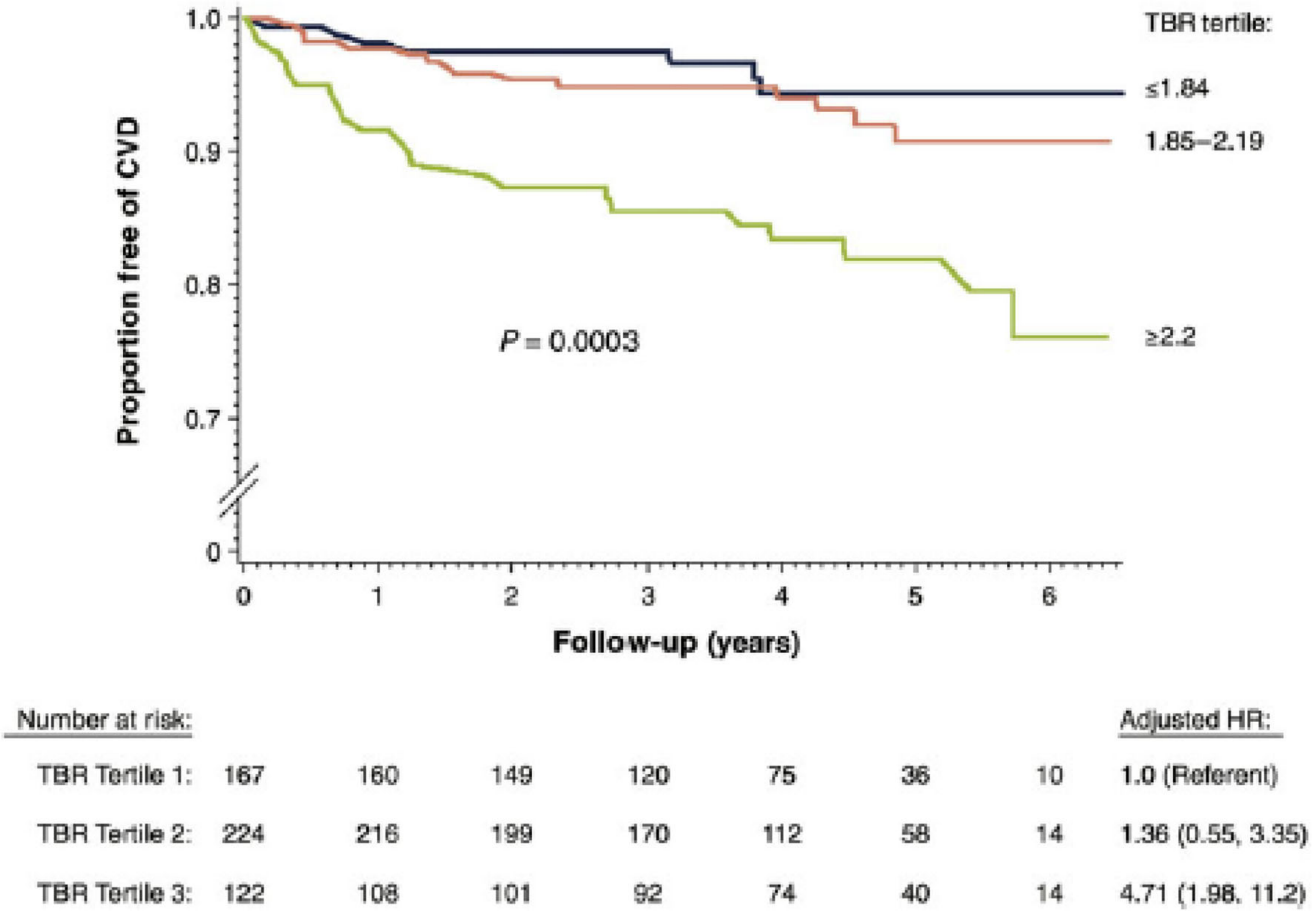
Proportion of participants who remained free from a cardiovascular event after an initial arterial ^18^F-fluorodeoxyglucose positron emission tomography scan. Arterial inflammation was determined as aortic FDG uptake corrected for background activity. Increased aortic inflammation on the baseline scan (a value within the highest tertile) associated with a higher risk of a subsequent cardiovascular event (median follow-up of 4.2 years). Reprinted with permission^1^

^18^F-flurodeoxyglucose (FDG) has been validated as a tracer for imaging vascular inflammation. The radio-labeled glucose analogue accumulates in glycolytically active cells and is used in clinical PET imaging to identify metabolically active tissue such as tumors and inflammatory foci. Both pre-clinical and human studies show that the uptake of FDG within the artery wall correlates with macrophage accumulation in arterial plaques. Several studies have shown that FDG-PET/CT imaging of arterial inflammation provides potent prognostic information to predict the risk of developing cardiovascular disease (CVD) events. Figueroa et al. demonstrated in cancer-free patients that aortic FDG uptake predicts incident CVD beyond traditional risk factors and coronary calcium scores. Furthermore, in patients with recent stroke secondary to carotid atherosclerosis, carotid inflammation independently predicts recurrent stroke. (Figures 2, 3)

## Role of FDG-PET/CT in monitoring novel therapies^1^

Measurement of arterial inflammation with FDG-PET/CT has been shown to be highly reproducible. As a result, it has been widely employed as a research tool to assess the impact of anti-atherosclerotic interventions. A non-randomized single center FDG-PET/CT study first reported a reduction in arterial inflammation following 3 months of low-dose statin therapy. A subsequent multi-center study demonstrated a graded anti-inflammatory action of high-dose vs low-dose atorvastatin. These PET/CT findings are in concert with the wealth of clinical endpoint trial data showing statins’ beneficial impact on CVD events. Similarly thiazolidinediones have been shown to reduce arterial inflammation in patients with type 2 diabetes mellitus and significantly reduce CVD events in this group.

Conversely, a cholesterol ester transfer protein modulator (dalcetrapib), lipoprotein-associated phospholipase A2 inhibitor (rilapladib), and P-38 MAP kinase antagonists (BMS-582949 and losmapimod) each failed to significantly reduce arterial inflammation in PET/CT trials (with the notable exception of post hoc exploratory endpoints). Congruently, large clinical endpoint trials have shown that compounds in the same drug class so far also have failed to reduce CVD events. Accordingly, FDG-PET/CT trials have the potential to quantify therapeutic efficacy for novel drug discovery and treatment strategies.

## Imaging of vascular calcification^1^,^2^

Vascular calcification is another important feature of atherosclerotic disease. Importantly, the associated risk attributable to vascular calcification diverges according to the size of the calcification. Microcalcification (deposits of calcification smaller than 50 μm) occurs predominantly in the fibrous cap and may contribute to mechanical plaque destabilization while macrocalcification may confer plaque stability^1^. While macrocalcification is detectable using conventional medical imaging, microcalcification cannot be detected on structural imaging. New techniques are required in order to detect and quantify the microcalcification process non-invasively in vivo.

^18^F-sodium fluoride (NaF) is a PET/CT radiotracer used in oncology to detect metastatic bone spread, where ^18^F-fluoride replaces the hydroxyl groups in hydroxyapatite. Within atheroma, microcalcification is largely felt to be an active process driven by osteoblast-like cells derived from vascular smooth muscle, though passive deposition of calcium phosphate deposits resulting from phosphate release from necrotic cells may also play a role. It is believed that NaF is incorporated into hydroxyapatite at the sites of active microcalcification sites in a similar manner to that seen in its oncology applications. This process is likely accelerated by inflammation, with plaque macrophage burden strongly associated with osteoblastic activity in the aorta of hyperlipidemic mice.^1^

**Figure 4:**
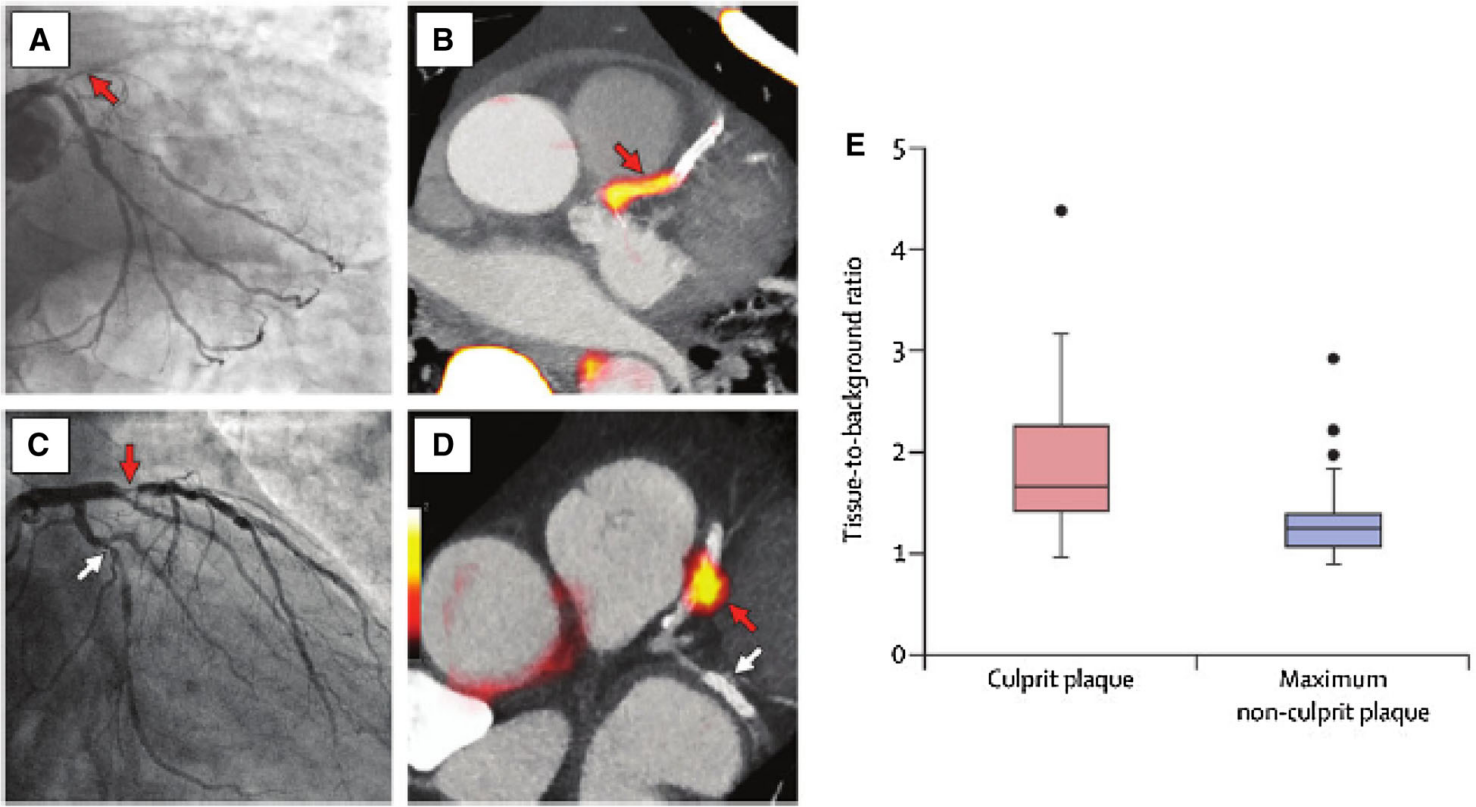
Coronary artery culprit lesions manifest increased ^18^F-sodium fluoride. ^18^F-sodium fluoride uptake was evaluated, using PET, in individuals who had a recent myocardial infarction. Two patients, (**A** and **C**), manifested culprit lesions in the left anterior descending artery (*red arrows* in **A** and **C**). ^18^F- sodium fluoride imaging in those same individuals (**B** and **D**) shows intense ^18^F-sodium fluoride activity in the same locations. **E** shows group mean data, which demonstrate elevated ^18^F-sodium fluoride activity in the culprit lesions compared to non-culprit vessels in the same individuals. Reprinted with permission^1^

Several studies have demonstrated NaF-PET/CT’s utility to detect microcalcification. Such studies show that NaF localizes to sites of active microcalcification (rather than simply reflecting the burden of calcification). Clinical studies have shown NaF uptake (measured by tissue-to-background ratios) is higher in symptomatic and high-risk atheroma compared to asymptomatic atheroma in both coronary and carotid arteries (Figure 4).

The development of techniques capable of imaging microcalcification has the potential to improve CVD risk, prognosis and guide the identification of new therapeutic strategies. Dweck et al. demonstrated that patients with higher vascular ^18^NaF uptake are more likely to have a clinical diagnosis of coronary artery disease and higher Framingham risk scores, with a correlation between NaF uptake and coronary artery calcium score, leading the authors to suggest NaF uptake could be used to refine CVD risk.

## Future considerations^1^

Several newer tracers have been evaluated for imaging high-risk biological features of atherosclerosis. ^68^Ga-DOTATATE targets the somatostatin receptor2, which is over-expressed in activated macrophages and endothelial cells responsible for inflammation and angiogenesis. This novel tracer has advantages over FDG for imaging coronary atheroma, since it is not taken up by myocardium. Additional tracers that target macrophages include ^11^C-PK11195 (targeting TSPO receptor), and ^18^F-FMCH (targeting macrophages cell membrane) have been shown in pre-clinical studies to localize to areas of active inflammation. Hypoxia within the plaque stimulates new vessel formation, increases LDL oxidation and promotes a pro-inflammatory response in macrophages. Hypoxia can be identified using ^18^F-fluoromisonidazole (^18^F-FMISO), a new tracer that identified cellular hypoxia as demonstrated by increased aortic uptake in an animal model fed an atherogenic diet. Neoangiogenesis can also be detected, using a tracer targeting integrin αvβ3expression (e.g., ^68^Ga-NOTA-RGD and ^18^F-Galacto-RGD).

As new therapies are developed (such as PCSK9 antagonists, along with other novel compounds drugs that may have more than minimal side effects), there will be a greater need for enhanced risk stratification. With such theoretical expansion of treatment options, there will be a need to better-match an individual’s risk against the cost and side effects of the new treatments. Additionally, imaging may be needed in order to assess whether the dose of the new therapies should be changed, or switched altogether, depending on changes seen on serial imaging, much the same way such decisions are made in oncologic diseases. Imaging biological features of atherosclerosis may contribute importantly to such enhanced risk assessment.

## Conclusion

PET/CT imaging provides outstanding opportunities to assess atherosclerotic plaque biology non-invasively. Current use of FDG and NaF enables the early detection of atherosclerotic plaque inflammation and microcalcification. Such an approach enhances our assessment of individual risk, and may help identify new therapies. Additional studies are needed to delineate the potential clinical role of such approaches especially as armamentarium to treat atherosclerotic disease growth, placing demands on better delineation of risk and a better understanding of treatment response.

## Electronic supplementary material

Below is the link to the electronic supplementary material.

Supplementary material 1 (PPTX 4009 kb)

## Abbreviations

CT: Computed tomography
CVD: Cardiovascular disease
FDG: Fluorodeoxyglucose
LDL: Low-density lipoprotein
MAP: Mitogen-activated protein
MI: Myocardial infarction
NaF: Sodium fluoride
PCSK9: Proprotein convertase subtilisin/kexin type 9
PET: Positron emission tomography
VCAM: Vascular cell adhesion molecules
