# Supplementary material for: Molecular imaging of atherosclerosis with integrated PET imaging
Source: J Nucl Cardiol. 2017 Jan 11;24(3):938–43. doi: 10.1007/s12350-016-0766-y (PMC5491686; doi:10.1007/s12350-016-0766-y)
Supplement: Supplementary file 1 — Supplementary material 1 (PPTX 4009 kb) [file 12350_2016_766_MOESM1_ESM.pptx]

## Slide 1
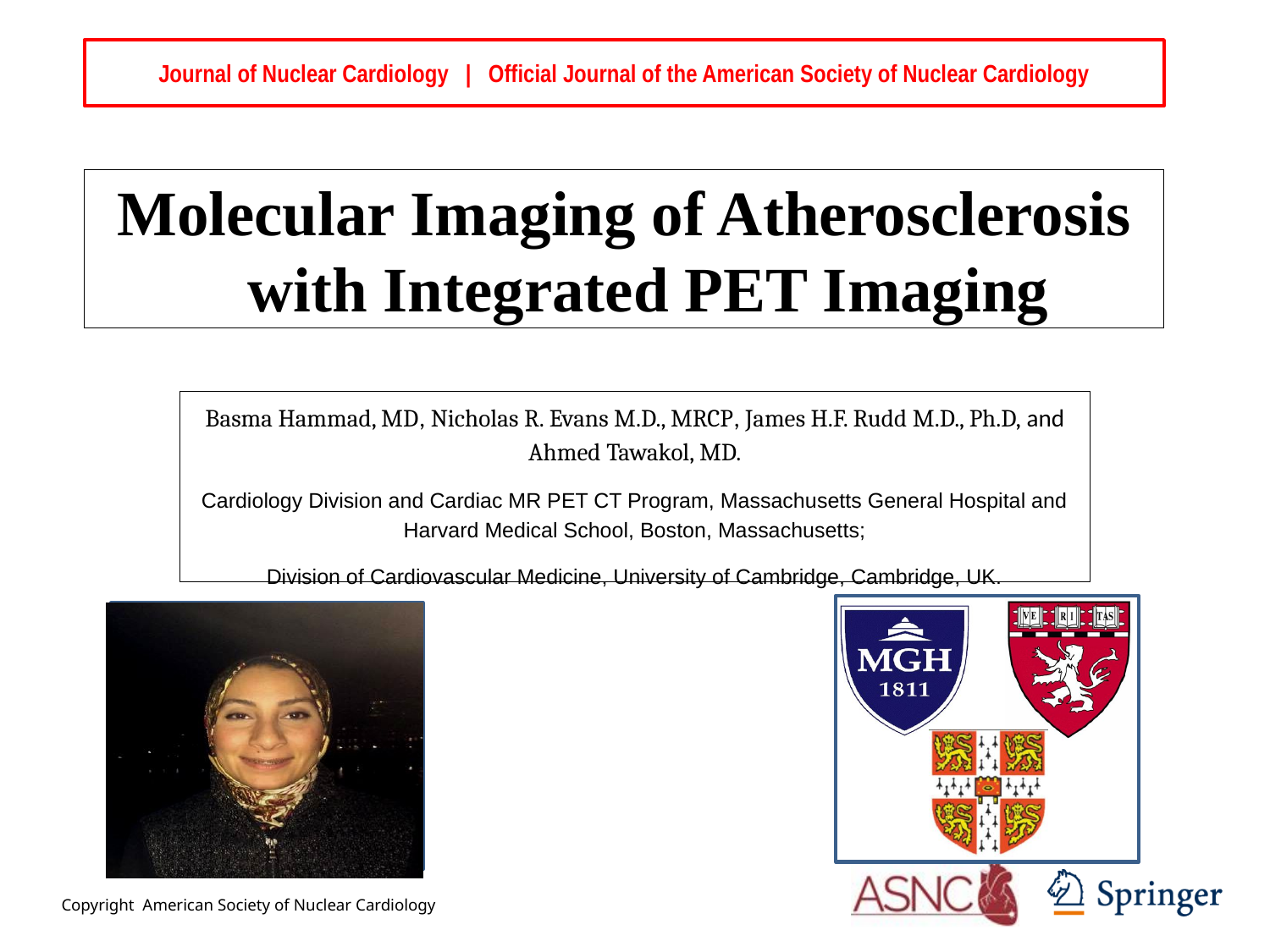

Journal of Nuclear Cardiology | Official Journal of the American Society of Nuclear Cardiology
# Molecular Imaging of Atherosclerosis with Integrated PET Imaging
Basma Hammad, MD, Nicholas R. Evans M.D., MRCP, James H.F. Rudd M.D., Ph.D, and Ahmed Tawakol, MD.
Cardiology Division and Cardiac MR PET CT Program, Massachusetts General Hospital and Harvard Medical School, Boston, Massachusetts;
Division of Cardiovascular Medicine, University of Cambridge, Cambridge, UK.
Head shot of author
required
Copyright American Society of Nuclear Cardiology

## Slide 2
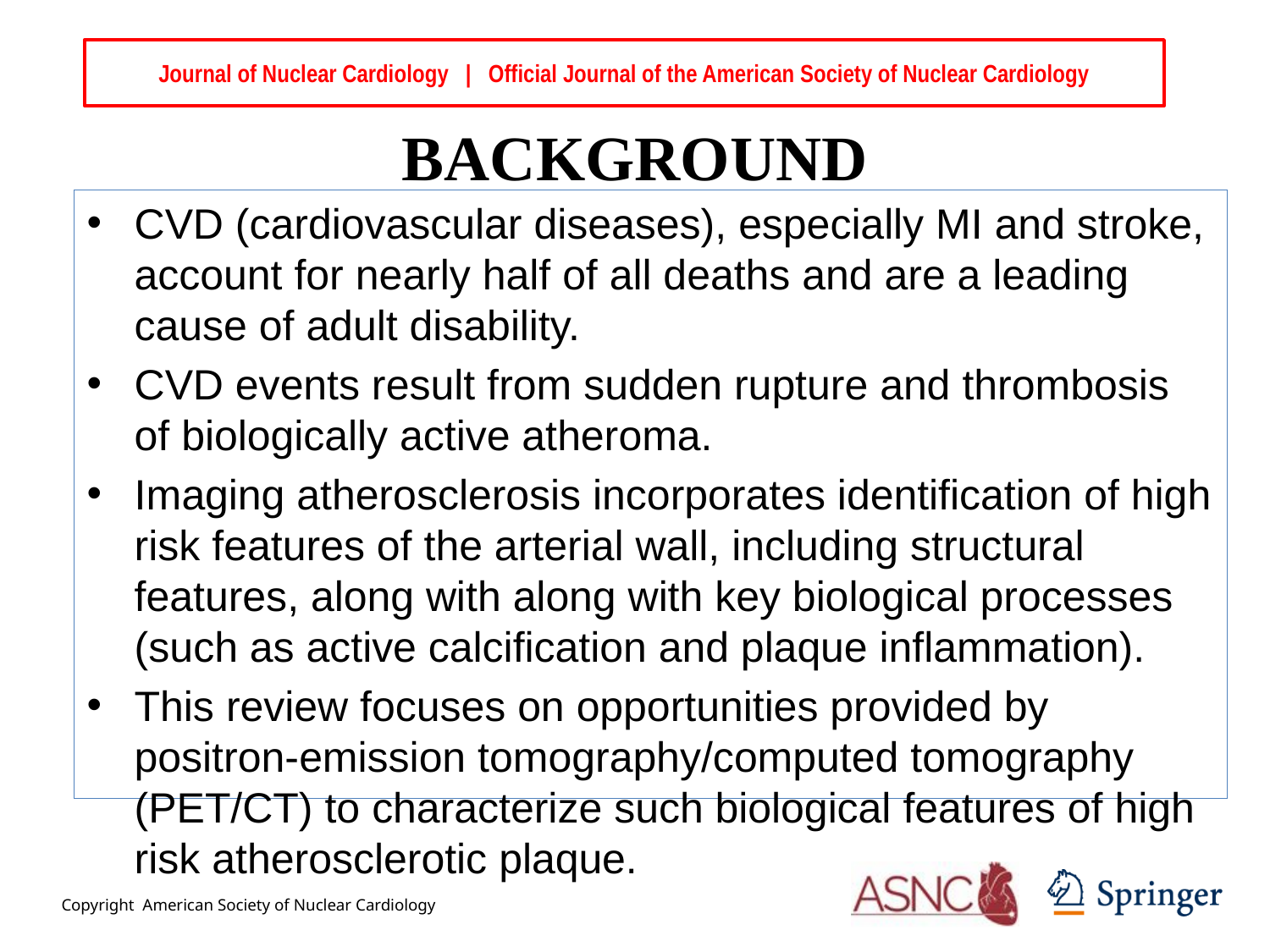

Journal of Nuclear Cardiology | Official Journal of the American Society of Nuclear Cardiology
# BACKGROUND
CVD (cardiovascular diseases), especially MI and stroke, account for nearly half of all deaths and are a leading cause of adult disability.
CVD events result from sudden rupture and thrombosis of biologically active atheroma.
Imaging atherosclerosis incorporates identification of high risk features of the arterial wall, including structural features, along with along with key biological processes (such as active calcification and plaque inflammation).
This review focuses on opportunities provided by positron-emission tomography/computed tomography (PET/CT) to characterize such biological features of high risk atherosclerotic plaque.
Copyright American Society of Nuclear Cardiology

## Slide 3
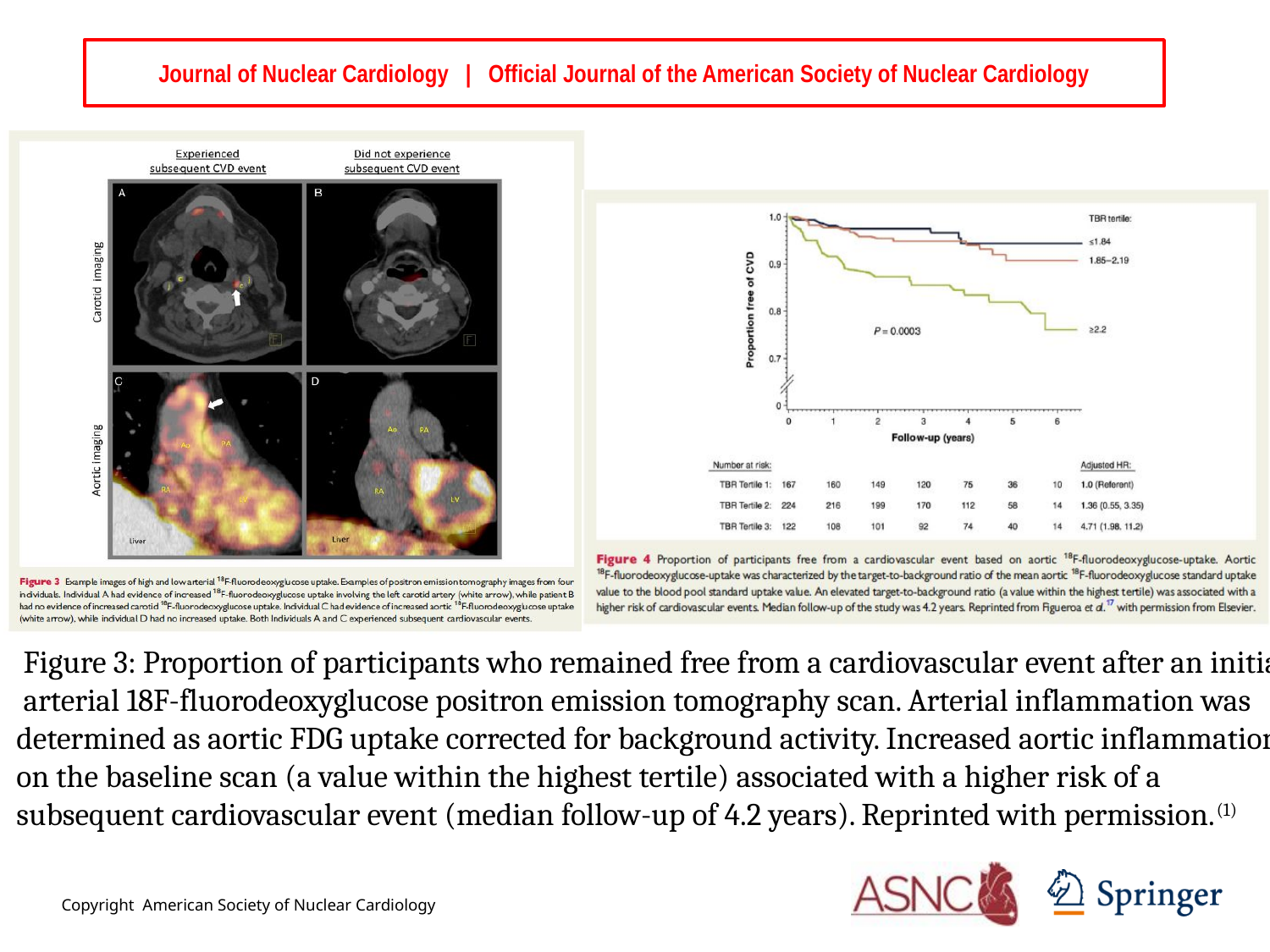

#
Journal of Nuclear Cardiology | Official Journal of the American Society of Nuclear Cardiology
 Figure 3: Proportion of participants who remained free from a cardiovascular event after an initial arterial 18F-fluorodeoxyglucose positron emission tomography scan. Arterial inflammation was determined as aortic FDG uptake corrected for background activity. Increased aortic inflammation on the baseline scan (a value within the highest tertile) associated with a higher risk of a subsequent cardiovascular event (median follow-up of 4.2 years). Reprinted with permission.(1)
Copyright American Society of Nuclear Cardiology

## Slide 4
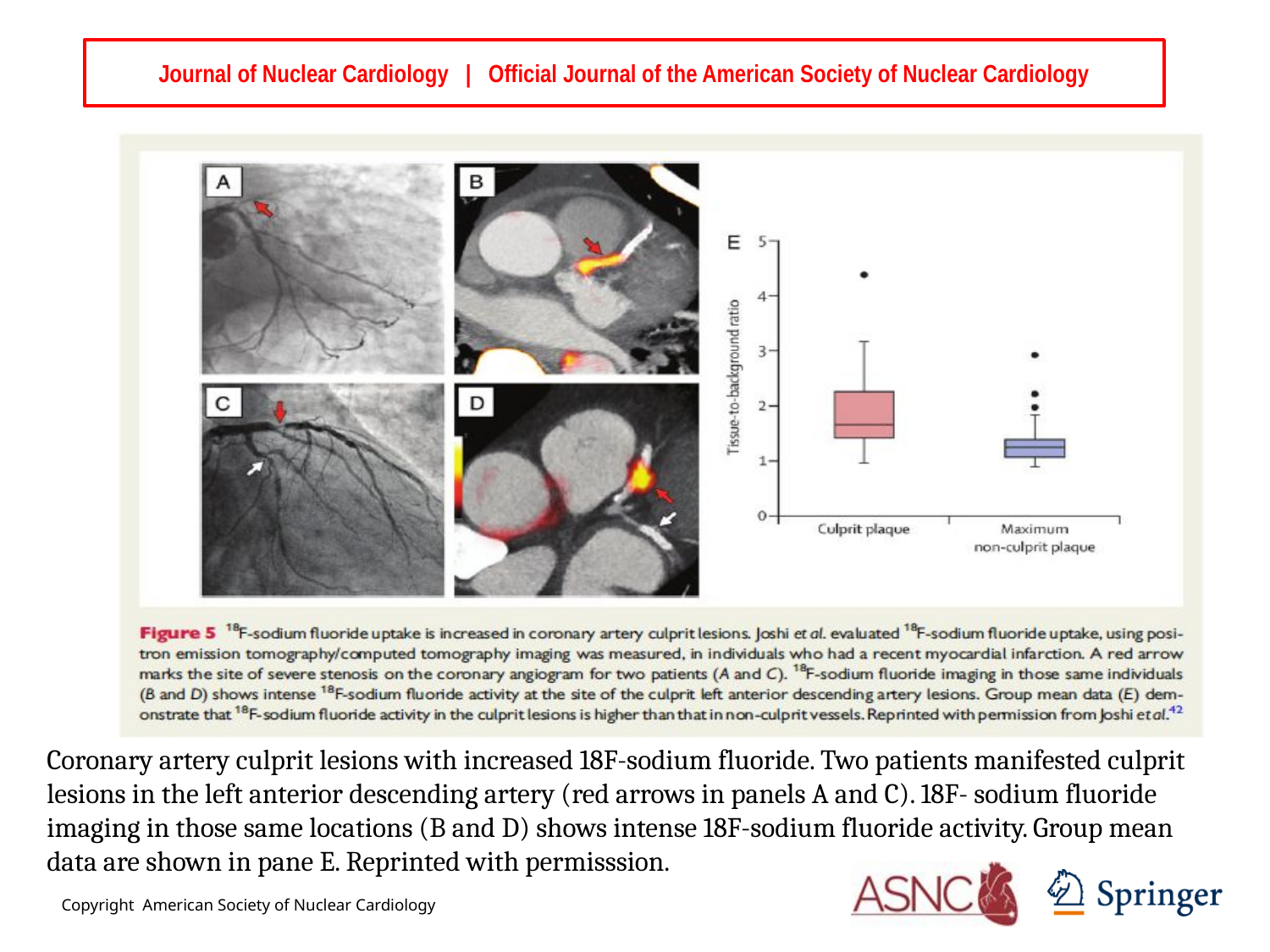

#
Journal of Nuclear Cardiology | Official Journal of the American Society of Nuclear Cardiology
Coronary artery culprit lesions with increased 18F-sodium fluoride. Two patients manifested culprit lesions in the left anterior descending artery (red arrows in panels A and C). 18F- sodium fluoride imaging in those same locations (B and D) shows intense 18F-sodium fluoride activity. Group mean data are shown in pane E. Reprinted with permisssion.
Copyright American Society of Nuclear Cardiology
